# Supplementary material for: A Computational Study on the Relation between Resting Heart Rate and Atrial Fibrillation Hemodynamics under Exercise
Source: PLoS One. 2017 Jan 11;12(1):e0169967. doi: 10.1371/journal.pone.0169967 (PMC5226796; doi:10.1371/journal.pone.0169967)
Supplement: S1 Table — Lumped-model parameters at baseline. P: pressure [mmHg], V: volume [ml], Q: flow rate [ml/s], ϑ: valve opening angle [rad]. E: elastance [mmHg/ml], C: compliance [ml/mmHg], R: resistance [mmHg s/ml], L: inductance [mmHg s2/ml], CQ: flow coefficient [ml/(s mmHg0.5)], K: valve coefficient. List of subscripts. la: left atrium, lv: left ventricle, ra: right atrium, rv: right ventricle, mi: mitral, ao: aortic, ti: tricuspid, po: pulmonary, un: unstressed, min: minimum, max: maximum, sas: systemic aortic sinus, sat: systemic artery, sar: systemic arterioles, scp: systemic capillary, svn: systemic vein, pas: pulmonary artery sinus, pat: pulmonary artery, par: pulmonary arterioles, pcp: pulmonary capillary, pvn: pulmonary vein, p: effect of pressure force, f: frictional action, b: velocity effect on the valve dynamics, due to blood motion, v: vortex effect on the valve dynamics. (DOCX) [file pone.0169967.s001.docx]

| **Left heart parameters** | | **Right heart parameters** | |
| --- | --- | --- | --- |
| **parameter** | **Value** | **parameter** | **Value** |
| CQ_ao_ | 350 ml/(s mmHg^0^*^.^*^5^) | CQ_po_ | 350 ml/(s mmHg^0^*^.^*^5^) |
| CQ_mi_ | 400 ml/(s mmHg^0^*^.^*^5^) | CQ_ti_ | 400 ml/(s mmHg^0^*^.^*^5^) |
| E_lv,max_ | 2.5 mmHg/ml | E_rv,max_ | 1.15 mmHg/ml |
| E_lv,min_ | 0.07 mmHg/ml | E_rv,min_ | 0.07 mmHg/ml |
| P_lv,un_ | 1 mmHg | P_rv,un_ | 1 mmHg |
| V_lv,un_ | 5 ml | V_rv,un_ | 10 ml |
| E_la,max_ | 0.25 mmHg/ml | E_ra,max_ | 0.25 mmHg/ml |
| E_la,min_ | 0.15 mmHg/ml | E_ra,min_ | 0.15 mmHg/ml |
| P_la,un_ | 1 mmHg | P_ra,un_ | 1 mmHg |
| V_la,un_ | 4 ml | V_ra,un_ | 4 ml |
| **Left heart valve parameters** | | **Right heart valve parameters** | |
| **parameter** | **Value** | **parameter** | **Value** |
| K_p,mi_, K_p,ao_ | 5500 ml/mmHg | K_p,ti_, K_p,po_ | 5500 ml/mmHg |
| K_f,mi_, K_f,ao_ | 50 s^-1^ | K_f,ti_, K_f,po_ | 50 s^-1^ |
| K_b,mi_, K_b,ao_ | 2 rad/(s ml) | K_b,ti_, K_b,po_ | 2 rad/(s ml) |
| K_v,mi_ | 3.5 rad/(s ml) | K_v,ti_ | 3.5 rad/(s ml) |
| K_v,ao_ | 7 rad/(s ml) | K_v,po_ | 7 rad/(s ml) |
| ϑ_max_ | 5/12 π rad | ϑ_max_ | 5/12 π rad |
| **Systemic parameters** | | **Pulmonary parameters** | |
| **parameter** | **value** | **parameter** | **Value** |
| C_sas_ | 0.064 ml/mmHg | C_pas_ | 0.162 ml/mmHg |
| R_sas_ | 0.003 mmHg s/ml | R_pas_ | 0.002 mmHg s/ml |
| L_sas_ | 0.000062 mmHg s^2^/ml | L_pas_ | 0.000052 mmHg s^2^/ml |
| P_sas,un_ | 1 mmHg | P_pas,un_ | 1 mmHg |
| V_sas,un_ | 25 ml | V_pas,un_ | 25 ml |
| C_sat_ | 1.28 ml/mmHg | C_pat_ | 3.42 ml/mmHg |
| R_sat_ | 0.05 mmHg s/ml | R_pat_ | 0.01 mmHg s/ml |
| L_sat_ | 0.0017 mmHg s^2^/ml | L_pat_ | 0.0017 mmHg s^2^/ml |
| P_sat,un_ | 1 mmHg | P_pat,un_ | 1 mmHg |
| V_sat,un_ | 775 ml | V_pat,un_ | 175 ml |
| R_sar_ | 0.44 mmHg s/ml | R_par_ | 0.0275 mmHg s/ml |
| R_scp_ | 0.4576 mmHg s/ml | R_pcp_ | 0.0385 mmHg s/ml |
| R_svn_ | 0.075 mmHg s/ml | R_pvn_ | 0.006 mmHg s/ml |
| C_svn_ | 20.5 ml/mmHg | C_pvn_ | 20.5 ml/mmHg |
| P_svn,un_ | 1 mmHg | P_pvn,un_ | 1 mmHg |
| V_svn,un_ | 3000 ml | V_pvn,un_ | 300 ml |

**S1 Table. Resting (1 MET) simulation parameters.** Lumped-model parameters at baseline. P: pressure [mmHg], V: volume [ml], Q: flow rate [ml/s], ϑ: valve opening angle [rad]. E: elastance [mmHg/ml], C: compliance [ml/mmHg], R: resistance [mmHg s/ml], L: inductance [mmHg s^2^/ml], CQ: flow coefficient [ml/(s mmHg^0^*^.^*^5^)], K: valve coefficient. List of subscripts. la: left atrium, lv: left ventricle, ra: right atrium, rv: right ventricle, mi: mitral, ao: aortic, ti: tricuspid, po: pulmonary, un: unstressed, min: minimum, max: maximum, sas: systemic aortic sinus, sat: systemic artery, sar: systemic arterioles, scp: systemic capillary, svn: systemic vein, pas: pulmonary artery sinus, pat: pulmonary artery, par: pulmonary arterioles, pcp: pulmonary capillary, pvn: pulmonary vein, p: effect of pressure force, f: frictional action, b: velocity effect on the valve dynamics, due to blood motion, v: vortex effect on the valve dynamics.
