# Supplementary material for: A Computational Study on the Relation between Resting Heart Rate and Atrial Fibrillation Hemodynamics under Exercise
Source: PLoS One. 2017 Jan 11;12(1):e0169967. doi: 10.1371/journal.pone.0169967 (PMC5226796; doi:10.1371/journal.pone.0169967)
Supplement: S2 Table — Cardiovascular parameters in Table 1 are varied, one at a time, by +5% and -5% for the two extreme conditions: 1 MET and 8 METs. Mean values of selected hemodynamic outputs (Psas, Ppvn, Ppas,dias, SV) are reported in SHR. (DOCX) [file pone.0169967.s002.docx]

|  | **1 MET (70 bpm)** | | | | **8 METs (130 bpm)** | | | |
| --- | --- | --- | --- | --- | --- | --- | --- | --- |
|  | **P_sas_ [mmHg]** | **P_pvn_**  **[mmHg]** | **P_pas,dias_**  **[mmHg]** | **SV**  **[ml]** | **P_sas_**  **[mmHg]** | **P_pvn_**  **[mmHg]** | **P_pas,dias_**  **[mmHg]** | **SV**  **[ml]** |
| **Table 1** | **97.13** | **10.35** | **11.12** | **74.25** | **131.19** | **14.53** | **17.41** | **103.85** |
| **E_lv,max_ -5%**  **E_lv,max_ +5%** | 96.23  97.96 | 10.44  10.28 | 11.20  11.05 | 73.54  74.91 | 130.77  131.56 | 14.54  14.52 | 17.41  17.41 | 103.51  104.15 |
| **E_rv,max_ -5%**  **E_rv,max_ +5%** | 96.87  97.36 | 10.32  10.38 | 11.09  11.14 | 74.00  74.48 | 131.08  131.29 | 14.52  14.55 | 17.39  17.42 | 103.75  103.94 |
| **C_sas_ -5%**  **C_sas_ +5%** | 97.16  97.10 | 10.36  10.35 | 11.12  11.11 | 74.27  74.23 | 131.23  131.16 | 14.54  14.53 | 17.41  17.40 | 103.88  103.82 |
| **C_sat_ -5%**  **C_sat_ +5%** | 97.69  96.57 | 10.43  10.28 | 11.20  11.04 | 74.68  73.81 | 131.99  130.39 | 14.62  14.44 | 17.52  17.30 | 104.50  103.20 |
| **R_sar_ -5%**  **R_sar_ +5%** | 95.80  98.45 | 10.35  10.35 | 11.12  11.12 | 74.71  73.80 | 129.43  132.93 | 14.56  14.51 | 17.44  17.37 | 104.19  103.50 |
| **R_scp_ -5%**  **R_scp_ +5%** | 95.74  98.50 | 10.35  10.35 | 11.12  11.12 | 74.73  73.78 | 129.36  133.00 | 14.56  14.51 | 17.45  17.37 | 104.21  103.49 |
| **C_svn_ -5%**  **C_svn_ +5%** | 98.71  95.60 | 10.50  10.21 | 11.28  10.96 | 75.48  73.06 | 132.23  130.16 | 14.65  14.42 | 17.55  17.27 | 104.69  103.02 |
| **R_pcp_ -5%**  **R_pcp_ +5%** | 97.24  97.02 | 10.36  10.34 | 11.03  11.21 | 74.34  74.16 | 131.31  131.06 | 14.55  14.52 | 17.27  17.55 | 103.96  103.74 |

**S2 Table. Sensitivity analysis of the AF simulations.** Cardiovascular parameters in Table 1 are varied, one at a time, by +5% and -5% for the two extreme conditions: 1 MET and 8 METs. Mean values of selected hemodynamic outputs (P_sas_, P_pvn_, P_pas,dias_, SV) are reported in SHR.
